# Evaluation of Hypoglycemic and Antioxidant Activities of Soybean Meal Products Fermented by *Lactobacillus plantarum* FPS 2520 and *Bacillus subtilis* N1 in Rats Fed with High-Fat Diet

**DOI:** 10.3390/metabo12050442

**Published:** 2022-05-14

**Authors:** Chung-Hsiung Huang, Chun-Lung Chen, Chen-Che Shieh, Shun-Hsien Chang, Guo-Jane Tsai

**Affiliations:** 1 Department of Food Science, National Taiwan Ocean University, Keelung 20224, Taiwan; huangch@mail.ntou.edu.tw (C.-H.H.); jeff8262@gmail.com (C.-L.C.); y82724ted2002@yahoo.com.tw (C.-C.S.); 2 Institute of Food Safety and Risk Management, National Taiwan Ocean University, Keelung 20224, Taiwan; lewis@mail.ntou.edu.tw; 3 Center for Marine Bioscience and Biotechnology, National Taiwan Ocean University, Keelung 20224, Taiwan

**Keywords:** antioxidant protection, *Bacillus*, hypoglycemia, fermented soybean meal, functional food, insulin resistance, *Lactobacillus*, probiotics

## Abstract

The hypoglycemic and antioxidant activities of *Lactobacillus plantarum* FPS 2520 and/or *Bacillus subtilis* N1 fermented soybean meal (SBM) in rats fed a high-fat diet (HFD) were investigated by assessing plasma glucose levels, insulin resistance, and oxidative stress-induced organ damage. Supplementation with FPS 2520- and/or N1-fermented SBM (500 and 1000 mg/kg of body weight per day) to HFD-induced obese rats for 6 weeks significantly down-regulated the concentration of plasma glucose during the oral glucose tolerance test (OGTT), as well as the concentration of fasting plasma glucose, insulin, and the value of the homeostasis model assessment of insulin resistance (HOMA-IR). In addition, plasma and hepatic levels of malondialdehyde (MDA) were alleviated in rats fed fermented SBM, especially SBM fermented by mixed strains. Moreover, fermented SBM treatment reduced HFD-exacerbated increases in plasma aspartate aminotransferase (AST), alanine aminotransferase (ALT), creatinine, and uric acid levels. Based on these results, we clearly demonstrate the effect of fermented SBM on improving insulin resistance and oxidation-induced organ damage. Therefore, it is suggested that fermented SBM has the potential to be developed as functional foods for the management of obesity-induced hyperglycemia and organ damage.

## 1. Introduction

Obesity has become a prominent public health issue, as it increases the risk of cardiovascular disease, diabetes, oxidative organ damage, non-alcoholic liver disease, and kidney failure [1,2]. It is well known that obesity increases the risk of developing type 2 diabetes, because obesity is a key contributor to the inducement of insulin resistance. Insulin resistance is not only a basic cause of type 2 diabetes mellitus, but it is also closely associated with many other pathophysiologic sequelae [3]. Obesity is reported to be related to an increase in endogenous lipid peroxides. Decreased production of malondialdehyde (MDA), which indicates lipid peroxidation, is significantly associated with weight loss, and has important implications for reducing obesity-related tissue and organ damage [4]. 

Soybean meal (SBM), a defatted soybean by-product of the food industry, contains high protein content and a variety of nutrients, such as isoflavones, dietary fiber, and oligosaccharides [5]. Soy isoflavones have many biological activities which significantly benefit metabolism and energy balance. For example, genistein treatment could raise energy depletion via up-regulating fatty acid catabolism in hepatocytes. Moreover, genistein also suppresses glucose uptake by adipocytes via down-regulating both glucose transporter 1 and glucose transporter 4 [6]. To improve bioavailability, soybean proteins can be hydrolyzed by microbial fermentation to harvest peptides and amino acids with a lower molecular weight and a higher rate of absorption. Moreover, microbial fermentation also converts isoflavone glycosides into aglycones, such as genistein and daidzein, which are more biologically active than their glucoside forms in regulating sugar and lipid metabolisms [7,8]. For example, previous studies screened *Lactobacillus plantarum* 128/2 and *Bacillus subtilis* natto NTU-18 with the activities of β-glucosidase production and isoflavone deglycosylation [9,10]. 

Moreover, bacteria *per se* may exert beneficial bioactivity. In HFD-fed C57BL6 mice, oral treatment with heat-killed *L. plantarum* OLL2712 (10^9^ CFU) for 12 weeks reduced the concentration of blood glucose in response to insulin [11]. With respect to microbial metabolites, oral treatment with *B. subtilis* SPB1 biosurfactant (10 mg/kg of body weight per day for one month) significantly decreased the concentration of blood glucose in diabetic rats [12]. On the other hand, some strains of *L. plantarum* and *B. subtilis* are reported to exhibit antioxidant activity [13,14]. Accordingly, it is hypothesized that *L. plantarum*- and/or *B. subtilis*-fermented products possibly exert hypoglycemic activity.

Although some studies investigated the impacts of microbial fermented soybean products and soybean-derived components of protein and genistein on plasma glucose and oxidative stress [8,15,16], limited information pertaining to the hypoglycemic and antioxidant effects of SBM, and even fermented SBM, is available. So far, most commercial fermented SBM products are designed to promote the growth and productivity of food-producing animals. In our previous report, fermentation of SBM by both single strain and mixed strains of *L. plantarum* FPS 2520 and/or *B. subtilis* N1 was established [17]. Oral administration of fermented SBM (500–1000 mg/kg of body weight per day) for 6 weeks reduced lipid accumulation and hyperlipidemia in high fat diet (HFD)-induced obese rats. Obesity is one of the major factors that elicits insulin resistance, hyperglycemia, and lipid peroxidation-associated liver and kidney damage. Therefore, the objective of the current study is to further explore the hypoglycemic and antioxidant activities of FPS 2520 and/or N1 fermented SBM products. The influence of fermented SBM on improving hyperglycemia, insulin resistance, lipid peroxidation, and impaired function of the liver and kidney, was investigated using the same model of HFD-induced obese rats [17].

## 2. Results

### 2.1. Daily Supplementation with Fermented SBM Ameliorated Hyperglycemia and Insulin Resistance in HFD-Induced Obese Rats

**Figure 1:**
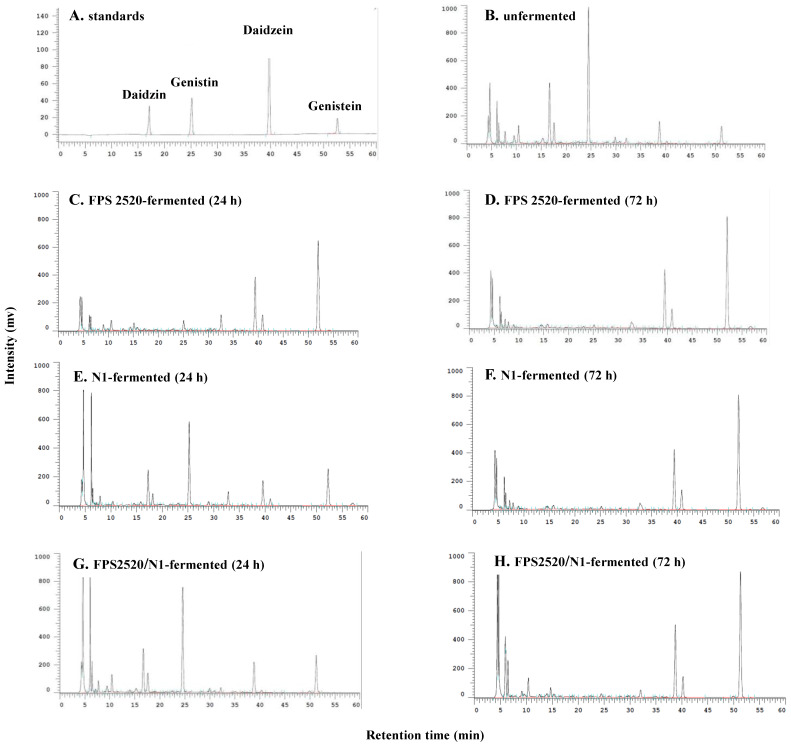
HPLC profiles of (**A**) isoflavone standard, (**B**) unfermented soy bean meal (SBM), SBM fermented by *Lactobacillus* sp. FPS 2520 for (**C**) 24 h, and (**D**) 72 h, SBM fermented by *Bacillus* sp. N1 for (**E**) 24 h, and (**F**) 72 h, and SBM fermented by mixed strains for (**G**) 24 h, and (**H**) 72 h.

**Figure 2:**
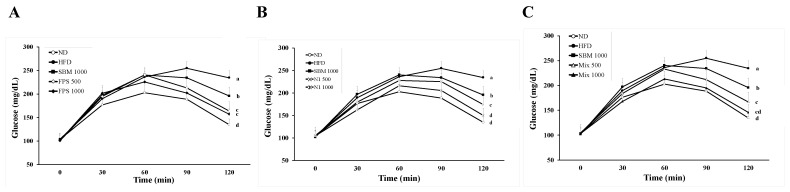
The concentration of plasma glucose in rats fed various fermented SBM products during the oral glucose tolerance test (OGTT). The rats were fed a HFD for 10 weeks, followed by various fermented SBM products for 6 weeks, as described in Materials and Methods. The blood samples were individually collected at the scheduled time points from rats fed SBM or products fermented with (**A**) *Lactobacillus* sp. FPS 2520 (FPS), (**B**) *Bacillus* sp. N1 (N1), or (**C**) mixed strains (Mix), after glucose treatment. Results are expressed as a mean ± standard deviation for each group of rats (*n* = 8). Different letters (a–d) indicate the statistical difference (*p* < 0.05) between each group.

**Figure 3:**
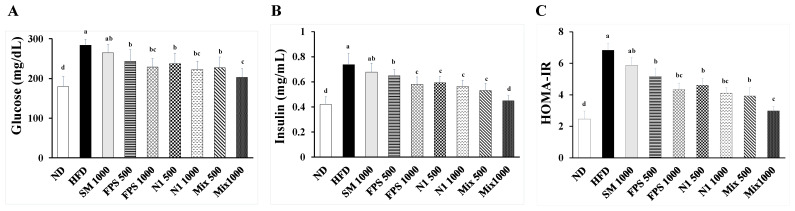
The impact of fermented SBM on fasting plasma glucose parameters. The rats were fed a HFD for 10 weeks, followed by various fermented SBM products for 6 weeks. The blood samples were individually collected after fasting for 12 h to determine the concentrations of (**A**) glucose and (**B**) insulin in plasma, and to calculate the (**C**) homeostasis model assessment equation-insulin resistance (HOMA-IR) value. Results are expressed as a mean ± standard deviation for each group of rats (*n* = 8). Different letters (a–e) indicate the statistical difference (*p* < 0.05) between each group.

As soy isoflavones have beneficial effects on improving metabolism [6], the contents of isoflavone glycosides and aglycones in SBM and fermented SBM were assessed. Compared to those in SBM, decreased levels of daidzin and genistin, and increased levels of daidzein and genistein were observed in fermented SBM (Figure 1). In both single strain- and mixed strains-fermentation, a longer period of fermentation resulted in a higher level of aglycones contained in the SBM (Figure 1). These results indicate that both FPS 2520 and N1 strains could effectively convert isoflavone glucosides to aglycones. To explore the effect of fermented SBM on attenuating obesity-associated hyperglycemia, OGTTs were conducted in HFD-induced obese rats after they were treated with various fermented SBM products for 6 weeks. As shown in Figure 2, ad libitum intake of an HFD for 10 weeks increased the level of plasma glucose at 30, 60, 90, and 120 min after the beginning of the OGTT, compared to intake of a normal diet, indicating successful induction of hyperglycemia using an HFD. Although non-fermented SBM also down-regulated the plasma level of glucose, lower levels of plasma glucose were observed in rats fed fermented SBM in a dose-dependent manner than in those fed non-fermented SBM. Moreover, the trend of the plasma level of fasting insulin observed in individual groups was similar to that of glucose (Figure 3A,B). Namely, fermented SBM products significantly down-regulated HFD-induced hyperglycemia and insulin resistance, with Mix 1000 being the most effective of these products. Because HOMA-IR is the index which more consistently predicts insulin resistance than others, we further calculated HOMA-IR based on fasting plasma glucose and insulin levels [18]. Concordantly, fermented SBM products treatment significantly reduced the value of HOMA-IR in a dose-dependent manner, with Mix 1000 being the most effective (Figure 3C).

### 2.2. Daily Fed with Fermented SBM Attenuated HFD-Induced Hepatic and Nephritic Oxidative Damage

**Figure 4:**
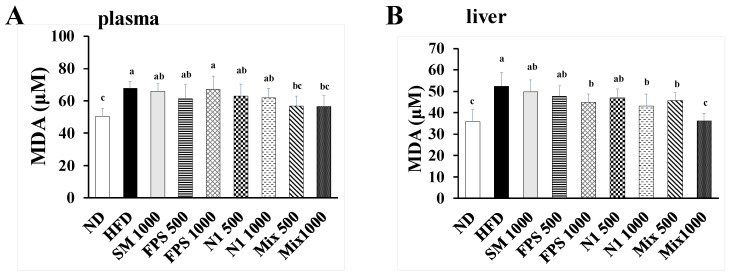
The levels of malondialdehyde (MDA) in the (**A**) plasma and (**B**) liver of rats fed with various fermented SBM products. The rats were fed an HFD for 10 weeks, followed by various fermented SBM products for 6 weeks. After sacrifice, the rats’ blood and liver samples were individually isolated. The concentration of MDA in the plasma and liver homogenates was determined. Results are expressed as a mean ± standard deviation for each group of rats (*n* = 8). Different letters (a–c) indicate the statistical difference (*p* < 0.05) between each group.

**Figure 5:**
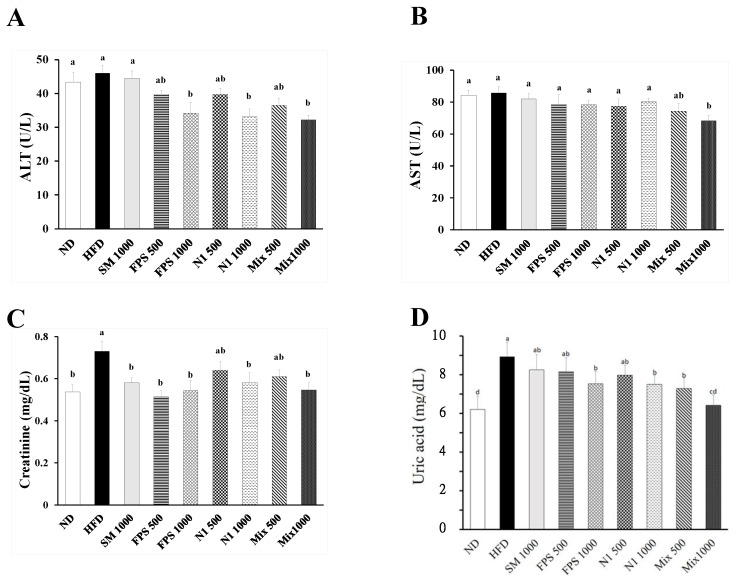
Evaluation of the impact of fermented SBM on hepatic and nephritic function. The rats were fed an HFD for 10 weeks, followed by various fermented SBM products for 6 weeks. The blood samples of rats were individually harvested to examine the plasma levels of (**A**) alanine aminotransferase (ALT), (**B**) aspartate aminotransferase AST, (**C**) creatinine, and (**D**) uric acid, using commercial kits. Results are expressed as a mean ± standard deviation for each group of rats (*n* = 8). Different letters (a–d) indicate the statistical difference (*p* < 0.05) between each group.

Since obesity is closely related to lipid peroxidation and oxidative damage, we further investigated the protective effects of fermented SBM products against HFD-induced lipid peroxidation and damage to the liver and kidney. As shown in Figure 4, the HFD treatment obviously elevated the level of MDA, an indicator of lipid peroxidation. However, fermented SBM products, especially products fermented by mixed strains, diminished the level of MDA in both plasma and liver homogenates. Because the plasma levels of alanine aminotransferase (ALT), aspartate aminotransferase (AST), creatinine, and uric acid are common biomarkers for evaluating liver and kidney function, respectively, these biomarkers were measured for preliminary examination of hepatic and nephritic damage. Although the levels of plasma ALT and AST were not diminished by an HFD, lower levels of ALT and AST were observed in rats fed with fermented SBM (Figure 5A,B), indicating that fermented SBM may have trophic effects on the liver. On the other hand, an HFD impaired kidney function was evidenced by raised concentrations of creatinine and uric acid (Figure 5C,D). Fermented SBM products significantly down-regulated concentrations of creatinine and uric acid (Figure 5C,D), revealing the protection provided by fermented SBM against obesity-associated kidney function impairment.

## 3. Discussion

Although the influence of soybean products on ameliorating obesity has been investigated in previous studies, the impacts of soybean products on obesity-associated hyperglycemia and oxidative stress are controversial. As our previous study demonstrated the anti-obesity effect of fermented SBM in HFD-fed rats, the objective of the current study is to explore the hypoglycemic and antioxidant effects of fermented SBM in the same animal model. Based on the data obtained from this study, dietary supplementation with fermented SBM for 6 weeks diminished the concentration of glucose in the plasma of HFD-fed rats during the OGTT, as well as the concentrations of fasting plasma glucose and insulin, and the value of HOMA-IR. In addition, the antioxidant effect of fermented SBM was evidenced by the attenuation of MDA concentration in both plasma and liver homogenates. In parallel, fermented SBM treatment also down-regulated the levels of ALT, AST, creatinine, and uric acid. Notably, the protection provided by fermented SBM products against hyperglycemia and oxidative damage was dose-dependent, with SBM fermented by mixed strains being the most potent product. 

The hypoglycemic and antioxidant effects of fermented soybean products have been previously reported. For example, a dietary supplement of cheonggukjang, a soybean paste fermented with *Bacillus licheniformis*, fed to HFD-treated mice for 13 weeks, decreased concentrations of blood glucose and insulin [19]. Extracts of cheonggukjang not only reduced plasma concentrations of fasting glucose and insulin, but also augmented the expression of antioxidant enzymes, including superoxide dismutase, catalase, and phospholipid hydroperoxide glutathione peroxidase, and protected against lipid peroxidation in HFD-treated mice [20]. In addition, the serum glucose, insulin, and lipid peroxidation of mice fed an HFD showed a dose-dependent decrease with the intake of natto, a fermented soybean product with *B. subtilis* [21]. *Bacillus subtilis*-fermented soybean consumption also improved glucose tolerance when it was employed with and without resistance exercise [22]. It has also been substantiated that long-term consumption of fermented soybean pastes protects HFD-fed obese mice against glucose intolerance, insulin resistance, and up-regulated mRNA expression of anti-oxidant enzymes [23]. Administration of okara, a soybean by-product, also reduces the serum level of insulin in HFD-fed mice [24]. Nevertheless, miso (fermented soybean paste) consumption had limited impact on the concentration of glucose and insulin serum of HFD-fed mice [25]. Compared to the above studies, this study further elucidates the hypoglycemic and antioxidant effects of SBM fermented by single strain and mixed strains. Our results clearly indicate that mixed strains-fermentation is a favorable process for SBM; of all these soy bean products, mixed strains-fermented SBM is the most potent against hyperglycemia and lipid peroxidation.

Although soy isoflavones, such as daidzein and genistein, are considered to be the major bioactive components contributing to the anti-obesity effects of soybean products, controversial results were observed in previous studies investigating the hypoglycemic and antioxidant effects of soybean-derived isoflavones. For example, soy isoflavones treatment improved insulin sensitivity among postmenopausal women [26]. Soy isoflavones administration normalized metabolic and immunological parameters via oxidative stress amelioration in female rats [27]. In male rats, soy isoflavones treatment (150 and 450 mg/kg of body weight per day for 4 weeks) showed a protective effect against HFD-induced testicular oxidative stress, cell apoptosis, and associated tissue damage [28]. As previously mentioned, soy isoflavone treatment supports the alleviation of exercise-induced oxidative stress, partially via enhancement of antioxidant enzyme activities [29]. A previous study also demonstrated that the free radical scavenging activity of genistein was higher than that of daidzein, indicating that genistein is a more promising antioxidant for the prevention of oxidative damage [30]. Furthermore, the synergistic amelioration effects of soybean soluble polysaccharides (a type of acidic polysaccharide containing 18% galacturonic acid) and genistein on dysglycemia, insulin resistance, and oxidative stress, were observed in mice fed an HFD [31]. However, Ricci et al. demonstrated that the use of isoflavone could not improve hyperglycemia in perimenopausal and postmenopausal non-Asian women [32]. Concordantly, treatment with daidzein or genistein for 6 months had a limited impact on dysglycemia and insulin resistance in Chinese women [33]. On the other hand, dietary soybean protein isolate could not improve blood glucose and insulin in HFD-fed mice [34]. Although our results show the effects of fermented SBM on down-regulating the levels of plasma MDA, AST, ALT, creatinine, uric acid, and hepatic MDA, further investigations should be conducted to understand whether fermented SBM improves the impaired hepatic and nephritic function via its antioxidant activity. For example, the data from histopathological examination, reactive oxygen species detection, and cellular apoptosis analysis will contribute to comprehensively clarifying the action mechanism of fermented SBM against hepatic and nephritic damages. Elucidation of the action mechanism of fermented SBM will help expand its indication to include fatty liver disease and gout disease. 

Except for isoflavones, the hypoglycemic activity of other bioactive soybean components, such as protein, peptide, dietary fiber, pectin, and pinitol, has been reported [35,36,37]. In addition, bacteria per se, employed for fermentation, may also contribute to the bioactivities of fermented SBM. It has been suggested that supplementation with probiotics has potential for improving type 2 diabetes, because probiotics are hypoglycemically active. For example, treatment with *L. plantarum* SS18–5 markedly improved the blood glucose of, and diminished oxidative stress in, diabetic rats [38]. Zhong et al. substantiated the protective activity of *L. plantarum* ZJUFB2 from insulin resistance via modulating enteric microbiota and bile acids in HFD-treated mice [39]. Inhibition of α-glucosidase and dipeptidyl peptidase-4, and modulation of gut microbiota are considered to be the potential mechanisms of the hypoglycemic effect of *L. plantarum* [40,41,42]. On the other hand, information pertaining to the hypoglycemic activity of *B. subtilis* is limited. However, the antidiabetic potential of metabolites from *B. subtilis* has been reported. Oral administration of *B. subtilis* SPB1 biosurfactant (10 mg/kg of body weight per day) to diabetic rats for one month could inhibit α-amylase and protect the pancreas’ β-cells [12]. Moreover, levan, an exopolysaccharide of fructose, and 1-Deoxynojirimycin, a strong α-glucosidase inhibitor, are considered to be major hypoglycemic components produced by *B. subtilis* [43,44]. Because a variety of soybean-derived components and bacterial metabolites may lead to hypoglycemic and antioxidant effects, a more complex phytochemical analysis will be required to understand the structure-activity of components in fermented SBM.

## 4. Materials and Methods

### 4.1. Chemicals, Reagents, Soybean Meal, and Rodent Diets

All chemicals were purchased from Sigma-Aldrich Chemical Co. (St. Louis, MO, USA) unless otherwise stated. Reagents for microbial culture were purchased from Becton, Dickinson and Company (Franklin, NJ, USA). Kits for glucose determination and biochemistry tests were purchased from Eagle Diagnostics (Cedar Hill, TX, USA) and Randox Laboratories, Ltd. (Crumlin, UK). Soybean meal was obtained from HUNG YANG Foods Co., Ltd. (Yunlin, Taiwan). The normal rodent diet (D12450H) and high fat diet (D12451i) were purchased from Research Diets Inc. (New Brunswick, NJ, USA).

### 4.2. SBM Fermentation Culture

The strains of *L. plantarum* FPS 2520 and *B. subtilis* N1 were isolated from the intestine of Pacific saury and a natto product (Typhula natto; Donan Hiratsuka Shokuhin Co., Ltd., Noboribetsu, Hokkaido, Japan), respectively, and the culture, storage, and preparation of bacteria for fermentation were conducted as described in the previous study [17]. Briefly, the strains of FPS 2520 and N1 were kept in glycerol-containing MRS and TSB (50%, *v*/*v*), respectively, at −80 °C. Before inoculation, the strains were subcultured twice in fresh broth without glycerol at 37 °C for 24 h. To prepare the SBM fermentation medium, 30 g of SBM and 240 mL of distilled water were mixed in a 500 mL volume baffled flask and then autoclaved. For single strain fermentation, a sterile 10% (*w*/*w*) SBM suspension was inoculated with either strain (10^4^ cfu/mL) and fermented at 37 ℃ for 72 h [17]. For mixed strains-fermentation, the sterilized SBM suspension was inoculated first with the N1 strain, fermented at 37 °C, 60 rpm for 24 h, and then the FPS2520 strain was added for another 24 h of fermentation. Finally, the broth was fermented at 45 °C with 60 rpm agitation for 24 h, then freeze-dried for animal testing.

### 4.3. Extraction and Analysis of Isoflavones

The fermented SBM samples were mixed with methanol (10%, *v*/*v*) and incubated in a 70 °C water bath for 30 min. After centrifugation at 18,000× *g* for 30 min, the supernatant was isolated and passed through a 0.22 μm pore size filter. Separation of isoflavones was performed using a Mightysil RP-18 GP column (Kanto chemical Co., Inc., Tokyo, Japan; 4.6 mm × 250 mm, 5 μm). Solvent A of the mobile phase was acetonitrile, and solvent B was 1% (*v*/*v*) acetic acid in water. Flow rate was set to 0.6 mL/min. The gradient elution was 85% solvent A for the first 5 min. The gradient elution was changed from 85 to 55% of solvent A for the following 5 to 45 min. Finally, the column was washed with solvent B for 5 min. HPLC chromatograms were detected using an HPLC detector (UV-1570, Jasco Analytical Instruments, Easton, MD, USA) at 260 nm. 

### 4.4. Animals and Experimental Design

Seventy-two five-week-old male Sprague Dawley (SD) rats were purchased from BioLASCO Taiwan Co., Ltd. (Taipei, Taiwan) and acclimatized for 1 week at the Terrestrial Animal Experimental Center of National Taiwan Ocean University (NTOU) with diet and water provided ad libitum. All animal experiments were conducted in accordance with the guidelines of the Terrestrial Animal Experimental Center of the NTOU under the approval protocol of the Institutional Animal Care and Use Committee of the NTOU, with approval number NTOU-107042. 

**Figure 6:**
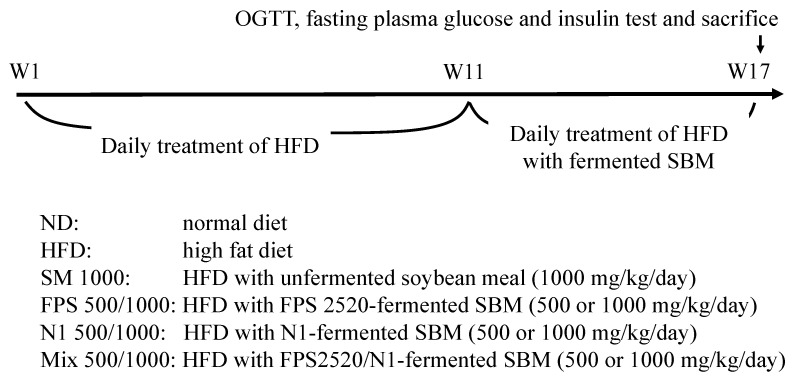
Protocols of fermented soybean meal (SBM) product administered to rats fed a high-fat diet (HFD). SD rats were fed a normal diet for 1 week, and then randomly divided into 9 groups (8 rats per group): normal diet group (ND); HFD control group; HFD with unfermented SBM (SM 1000; 1000 mg/kg); HFD with FPS 2520-fermented SBM (FPS 500 and FPS 1000; 500 and 1000 mg/kg); HFD with N1-fermented SBM (N1 500 and N1 1000; 500 and 1000 mg/kg); and HFD with FPS2520/N1-fermented SBM (Mix 500 and Mix 1000; 500 and 1000 mg/kg). All rats, except those in the ND group, were fed a HFD for 10 weeks, and then various fermented SBMs were orally administered to rats daily for the following 6 weeks. Oral glucose tolerance tests were conducted by orally administering glucose (1.5 g/kg of body weight) to rats, and then blood sampling at 0, 30, 60, 90, and 120 min. Moreover, blood samples were collected after food deprivation of 12 h, and the rats were sacrificed to harvest liver samples for further analysis.

The experimental design and protocol of this animal study was conducted in accordance with our previous study demonstrating the anti-obesity effect of fermented SBM in HFD-fed rats [17]. As shown in Figure 6, rats were fed a normal diet for 1 week (from day 1 to day 7), and then randomly divided into 9 groups (8 rats per group): normal diet group (ND); HFD control group; HFD with unfermented SBM (SM 1000; 1000 mg/kg) group; HFD with FPS 2520-fermented SBM (FPS 500 and FPS 1000; 500 and 1000 mg/kg) group; HFD with N1-fermented SBM (N1 500 and N1 1000; 500 and 1000 mg/kg) group; and HFD with FPS2520/N1-fermented SBM (Mix 500 and Mix 1000; 500 and 1000 mg/kg) group. All rats, except those in the ND group, were fed an HFD for 10 weeks (from day 8 to day 77); various fermented SBMs were orally administered to rats daily for the following 6 weeks (from day 78 to day 119). On day 119, oral glucose tolerance tests were conducted by orally administering glucose (1.5 g/kg of body weight) to rats, and then blood sampling at 0, 30, 60, 90, and 120 min. Moreover, blood samples were collected after food deprivation of 12 h, and the rats were sacrificed to harvest liver samples on day 120. The blood samples and liver samples were collected individually (i.e., 8 samples per group) at the anticipated time points for the OGTT, fasting glucose and insulin measurements, biochemistry tests, and MDA determination.

### 4.5. Biochemistry Test for the Plasma Levels of Glucose, Insulin, AST, ALT, Creatinine and Uric Acid

The plasma levels of glucose, insulin, AST, ALT, creatinine, as well as uric acid levels were determined using glucose detection kits, insulin ELISA kits, and Enzymatic Kits (Randox Laboratories Limited, Crumlin, UK), respectively, according to the manufacturers’ instructions. The HOMA-IR was calculated using the following equation [18]:

HOMA-IR = fasting insulin (μIU/mL) × fasting glucose (mmol/mL)/22.5(1)

### 4.6. Evaluation of Malondialdehyde (MDA) Levels in the Plasma and Liver

Based on the protocol of Uchiyama and Mihara [45], the samples were homogenized in 1.15% KCl solution, then mixed with 2-thiobarbitutric acid, followed by incubation in a boiling water bath for 45 min, and then cooled. After centrifugation at 1600× *g*, 4 ℃ for 10 min, the samples were kept at room temperature for 30 min, and then the supernatants were collected and measured using a Luminescence Spectrometer (Hitachi, F2000, Tokyo, Japan) with excitation at 520 nm and emission at 535 nm.

### 4.7. Statistical Analysis

SPSS Version 12.0 (SPSS Inc., Chicago, IL, USA) and one-way analysis of variance (ANOVA) were employed to analyze the data and to determine the statistical differences between each group, respectively, with the level of significance set at *p*  <  0.05. Multiple comparisons of means were analyzed using Duncan’s multiple range test. The results are expressed as mean ± standard deviations.

## 5. Conclusions

This is the first study to demonstrate and compare the effects of SBM fermented by single strain and mixed strains of *L. platarum* FPS 2520 and *B. subtilis* N1 on attenuating obesity-associated hyperglycemia and oxidative damage. These data also reveal that the product fermented by mixed strains was the most potent fermented SBM product. The levels of isoflavone glycosides and aglycones in fermented SBM products were comparable; therefore, it is suggested that isoflavone and microbial metabolites produced by both strains exert a synergistic effect in alleviating hyperglycemia and lipid peroxidation. How the interaction between microbial fermentation metabolites and SBM-derived bioactive components may contribute to these effects remains elusive. This issue is intriguing and warrants further investigation. Owing to the promising activity of fermented SBM against glucose intolerance, insulin resistance and oxidative stress, we suggest that fermented SBM products can prospectively be developed as functional foods or additives for preventing obesity-associated hyperglycemia and oxidative damage.

## Data Availability

Data for this study are available upon request from the corresponding author.
